# A human activity recognition method based on Vision Transformer

**DOI:** 10.1038/s41598-024-65850-3

**Published:** 2024-07-03

**Authors:** Huiyan Han, Hongwei Zeng, Liqun Kuang, Xie Han, Hongxin Xue

**Affiliations:** 1 School of Computer Science and Technology, North University of China, Taiyuan, 030051 China; 2 Shanxi Key Laboratory of Machine Vision and Virtual Reality, Taiyuan, 030051 China; 3 Shanxi Vision Information Processing and Intelligent Robot Engineering Research Center, Taiyuan, 030051 China

**Keywords:** Human activity recognition, Skeleton data, Spatio-temporal, ViT, Computer science, Applied mathematics

## Abstract

Human activity recognition has a wide range of applications in various fields, such as video surveillance, virtual reality and human–computer intelligent interaction. It has emerged as a significant research area in computer vision. GCN (Graph Convolutional networks) have recently been widely used in these fields and have made great performance. However, there are still some challenges including over-smoothing problem caused by stack graph convolutions and deficient semantics correlation to capture the large movements between time sequences. Vision Transformer (ViT) is utilized in many 2D and 3D image fields and has surprised results. In our work, we propose a novel human activity recognition method based on ViT (HAR-ViT). We integrate enhanced AGCL (eAGCL) in 2s-AGCN to ViT to make it process spatio-temporal data (3D skeleton) and make full use of spatial features. The position encoder module orders the non-sequenced information while the transformer encoder efficiently compresses sequence data features to enhance calculation speed. Human activity recognition is accomplished through multi-layer perceptron (MLP) classifier. Experimental results demonstrate that the proposed method achieves SOTA performance on three extensively used datasets, NTU RGB+D 60, NTU RGB+D 120 and Kinetics-Skeleton 400.

## Introduction

Human activity recognition is an important research subject in the field of computer vision, whose main task is to identify movements of human body in various visual scene. The emergence of image sensors and immense computational capabilities of AI make human activity recognition possible. In recent years, methods based on deep learning have better recognition accuracy and generalization performance than traditional methods, but some human behaviors are more similar, which makes the recognition rate low.

**Figure 1:**
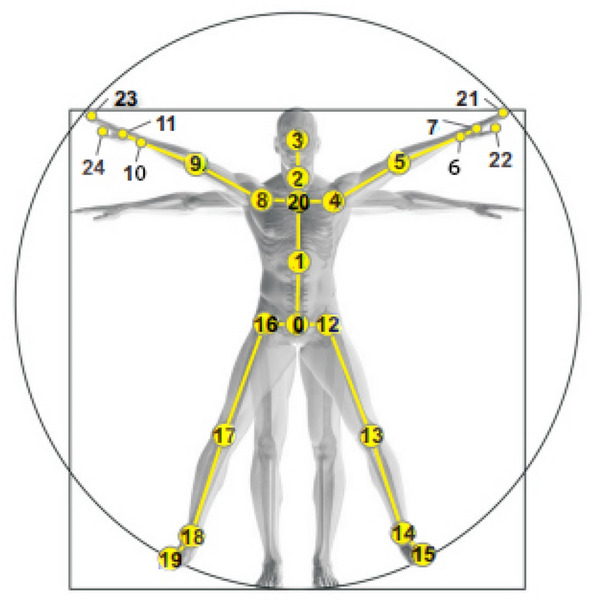
Human skeleton joint location information.

The human body is a complex structure with multiple degrees of freedom, and a single two-dimensional image/video cannot provide an unique or stable solution. In contrast, 3D skeleton data provides body pose and movement information directly, exhibits better invariance (including scale, camera viewpoint and noise of background interference), enabling it a more accurate representation of human activity without ambiguity, as shown in Fig. 1. Meanwhile, depth sensors (Kinect), availability of pose estimation algorithms (TransPose^1,2^, TokenPose^3^) and large-scale standard datasets^4,5^ make the skeleton-based HAR research extensive.

Originally, the researchers extracted manual features from skeleton sequences^6,7^. With the development of deep learning, various network architectures have been used to process this type of data. Recurrent neural networks (RNNs) are employed to compute the temporal information^8,9^. Convolutional neural networks (CNNs) denote skeleton data as pseudo-images (belongs to Euclidean space)^10,11^. GCNs have been widely used because its strong ability to capture intrinsic relationships between nodes (joints) in non-Euclidean space (skeleton graph). ST-GCN is the first GCN algorithm working on 3D skeleton data, it utilizes spatio-temporal graph convolution kernels for down-sampling in order to achieve classification results^12^. 2s-AGCN^13^ generates dynamic skeleton graphs using an adaptive attention mechanism. AGCN algorithm enhances the dynamic graph structure of the inference process based on ST-GCN^14^. CA-GCN relies on context information (capture feature of each vertex by integrating information of all other vertices and long range dependencies among joints) to output classification results^15^. SS-GCN constructs a novel graph network based on both spatio-temporal information and spectral-domain information^16^. CTR-GCN dynamically learn different topologies and effectively aggregate joint features in different channels for skeleton-based action recognition^17^. HD-GCN decomposes every joint node into several sets to extract major structurally adjacent and distant edges, and uses them to construct an HD-Graph containing those edges in the same semantic spaces of a human skeleton^18^. LKA-GCN enlarges the receptive field and improves channel adaptability without increasing too much computational burden^19^. DeGCN learns the deformable sampling locations on both spatial and temporal graphs, enabling the model to perceive discriminative receptive fields^20^. In DS-GCN, the joints and edge types are encoded in the skeleton topology in an implicit way, the joints type-aware adaptive topology and the edge type-aware adaptive topology are proposed^21^.

Although GCN-based methods have made significant progress, these methods still have two challenges: (1) how to model remote (long-distance) dependencies between joints more accurately, thereby alleviating the over-smoothing problem caused by stack graph convolutions. (2) How to improve robustness and semantics correlation to capture the large movements between time sequences. Therefore, the motivation of our work is to offer a feasible and effective approach for addressing these aforementioned limitations, which can be summarized as follows:

### Motivation 1

ViT model applies the Transformer architecture in image recognition^22^, it interprets an image as a sequence of patches and processes it by a standard Transformer encoder as used in NLP. This simple, yet scalable strategy works surprisingly well when coupled with pre-training on large datasets. ViT matches or exceeds SOTA methods on many image classification datasets, whilst being relatively cheap to per-training. Both the lower layers and the high layers of the ViT model structure can have a large field of view, global feature information can be obtained in initial layer, so it can well guarantee the global and local features integrity and can resolve the over-smoothing problem caused by stack graph convolutions. Skip connections have a huge impact on the propagation and representation of feature, and so it can capture semantics correlations of joints between time sequences. ViT retains location information while transmitting feature information. By leveraging the advantages of ViT and addressing the shortcomings of GCN-based methods, we innovatively apply ViT in this kind of skeleton data with spatial and temporal characteristics.

### Motivation 2

The inputs of both ViT and its other applications are 2D images, ViT can not be directly used in 3D skeleton data. The adaptive matrix is integrated in adjacency matrix of 2s-AGCN in the graph convolution, so that the model can adjust the topology of the graph adaptively and adapt to different input data. This innovative design greatly improves the recognition accuracy of skeleton data. However, the ability of capturing hidden data is insufficient and it is easy to cause gradient disappearance in product operation. We enhance adaptive graph convolutional layer (AGCL) in 2s-AGCN by replacement two embedding functions and normalization by a scale factor of Scaled Dot-Product Attention of trainable adjacency *C*_*k*_, namely eAGCL, thus it can automatically learn the connection strength problem between joints in the sample data and solve the gradient disappearance problem.

### Our contributions

In summary, our work makes three major contributions:

1. For the first time, ViT is applied to skeleton 3D data and put forward a human behavior recognition method HAR-ViT.
2. The position encoder in ViT is rewritten to order the non-sequenced information (skeleton data) and reduce the idle spatial coding information.
3. We propose eAGCL model based on AGCL in 2s-AGCN, improve utilization of spatial features of our network model.

At last, our HAR-ViT exhibits competitive performance on two public, authoritative recognition datasets (NTU-RGB+D 60 , NTU-RGB+D 120 and Kinetics-Skeleton 400) and outperforms some SOTA methods to a certain extent.

The rest of paper is organized as follows. Section "Related work" introduces the related work. Section "Materials and methods" introduces the components of our new method proposed in this paper. The ablation study and the comparison with the SOTA methods are are shown in Section "Experiments and results". Section "Conclusions" concludes the paper.

## Related work

This work aims to design a more robust solution for skeleton-based action recognition tasks inspired by ViT. This section discusses some works based on ViT and AGCL in 2s-AGCN.

### ViT

ViT attains excellent image classification results compared to SOTA convolutional networks while requiring substantially fewer computational resources to train. It shows that the reliance on CNNs is not necessary and a pure transformer applied directly to sequences of image patches can perform very well, especially when train with large scale training sets. In addition, the model pre-trained on large-scale datasets can also achieve better performance than CNN when migrating to medium or small datasets.

The lowest level of ViT allows the model to have larger windows through the self-attention mechanism. In the shallow layer, the model gradually acquires local and global characteristics while at the deep layer, the model has acquired the characteristics of a global view from the very beginning. Skip links play a important role on the propagation and representation of feature, and if they are removed, the accuracy of the model decreases by about 4%. The similarity between the input image and the feature map of the last layer in ViT is very high and this indicates that ViT retains location information while propagating feature information.

Pyramid ViT implements a variable self-attention mechanism through a space-reduction attention mechanism and is applied to ViT models to overcome square complexity in the attention mechanism^23^. A hierarchical ViT model with sliding windows is used by Swin Transformer, a local self-attention mechanism is applied to non-overlapping windows based on its position, thus forming a hierarchical feature representation in the next level and finally integrate these features^24^. DINO is a self-supervised training framework proposed by Meta's AI team based on ViT, which can be trained on large-scale unlabeled data and obtain robust feature representation, even without a fine-tuned linear layer^25^. Scaling ViT has won first place in ImageNet's recognition result, it is proposed by Google Brain team by scaling up ViT model with 2 billion parameters^26^. SegFormer proposal by Nvidia focuses on the componentization of the system and adopts a simple MLP decoding model without requiring position encoder^27^.UNETR (Unet+ViT) utilizes a transformer as the encoder to learn sequence representations of the 3D medical images and effectively capture the global multi-scale information, the transformer encoder is directly connected to a decoder via skip connections at different resolutions to compute the final semantic segmentation output, it achieves preferable semantic segmentation^28^.We apply ViT to 3D skeleton human behavior recognition on account of its excellent performance on 2D and 3D image process and propose a method namely HAR-ViT.

### AGCL in 2s-AGCN

**Figure 2:**
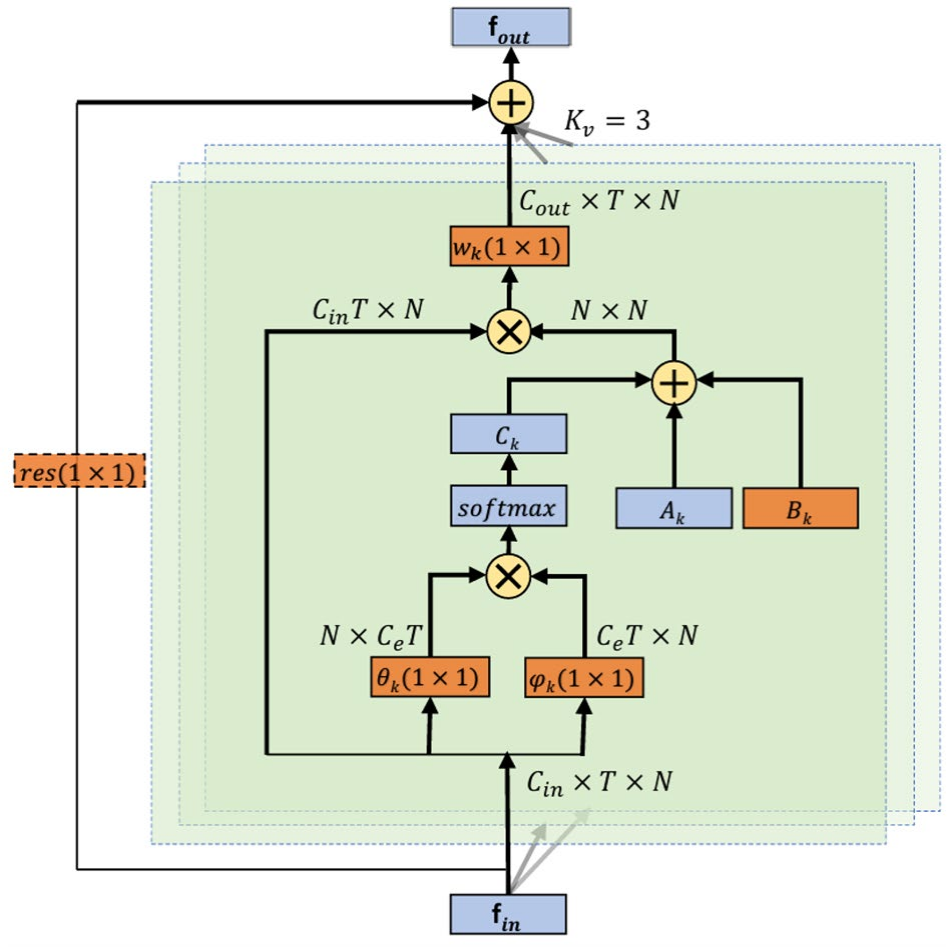
The network structure of AGCL^13^.

The AGCL serves as the fundamental neural network layer of 2s-AGCN, enabling an end-to-end learning approach to optimize skeleton data. The network of AGCL is illustrated in Fig. 2. Its topology and network parameters are designed to enhance flexibility by accommodating unique data graphs for different layers and samples. Additionally, it is constructed as a residual branch to ensure the stability of the original model.

The graph convolution rule of AGCL is shown in Eq. (1), where *f*_*out*_ and *f*_*in*_ denote the output and input of the network respectively. *K*_*v*_ represents the kernel size of the spatial dimension, which is set to 3. *k* denotes the position of kernel, while *W*_*k*_ signifies the operation of a 1 × 1 convolution. The adjacency matrix is divided into three parts: *A*_*k*_, *B*_*k*_, and *C*_*k*_. *A*_*k*_ denotes the fixed connectivity pattern among human joints. *B*_*k*_ demonstrates the connection strength of two joints. The elements of *B*_*k*_ are parameterized and optimized together with the other parameters in the training process. *C*_*k*_ is a data dependency graph that learns a unique graph for each sample. To determine the existence and strength of connections between two joints, it uses the normalized embedding Gaussian function to calculate the similarity between two joints (Eq. 2), while utilizing dot product to measure their similarity in the embedding space. By applying two embedding functions *θ*(*v*_*i*_) and *φ*(*v*_j_) to the feature map to rearrange them into matrices with dimensions N × T and T × N respectively, then multiply these matrices together to obtain an N × N similarity matrix *C*_*k*_. Each element of *C*_*k*_ represents the similarity between corresponding joints $\left({v}_{i}, {v}_{j}\right)$.

1 $$ {{\text{f}}_{{{\text{out}}}}  = \sum\limits_{{{\text{k}} = 1}}^{{{\text{K}}_{{\text{v}}} }} {{\text{W}}_{{\text{k}}} {\text{f}}_{{{\text{in}}}} } \left( {{\text{A}}_{{\text{k}}}  + {\text{B}}_{{\text{k}}}  + {\text{C}}_{{\text{k}}} } \right)} $$

2 $$\begin{array}{c}f\left({v}_{i}, {v}_{j}\right)=\frac{{e}^{{\theta \left({v}_{i}\right)}^{T}\varphi \left({v}_{j}\right)}}{{\sum }_{j=1}^{N}{e}^{{\theta \left({v}_{i}\right)}^{T}\varphi \left({v}_{j}\right)}}\end{array}$$

The residual connection of *B*_*k*_ and *C*_*k*_ in the model enhances its flexibility and stability without sacrificing the original performance. However, exponential order computing of Gaussian function ignores hidden information between samples and fails to sufficiently calculate the similarity between the joints. Moreover, its computational cost is very expensive and prone to gradient disappearance. We improve the accuracy of similarity and decrease computing costs by replacement the two embedding functions (*θ* and *φ*) and normalization by a scale factor.

## Materials and methods

**Figure 3:**
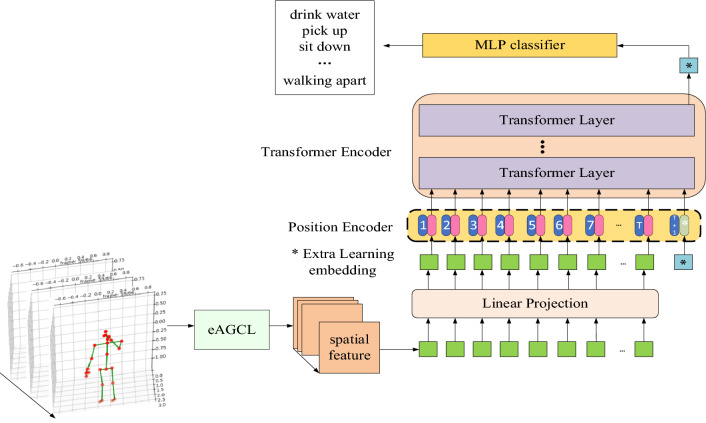
The overall framework of HAR-ViT.

In this paper, we propose an end-to-end HAR-ViT algorithm that creatively applies ViT to the field of human activity recognition. The overall framework of the model is illustrated in Fig. 3.

To address the limitation of ViT not being able to process 3D skeleton data, this study enhances the AGCL in 2s-AGCN. This enhancement enables extraction of spatial features encompassing connection relationships and strengths between joints. These features are then sorted along the timing axis before being transformed into feature vectors through linear projection.

In order to capture temporal features, learnable embedding vectors are added to the end of the feature vectors, while a position encoder is integrated with them to provide temporal information. Subsequently, these fused feature vectors serve as input for transformer encoder where multiple Transformer Layer networks extract temporal feature by means of similarity computation. Finally, all temporal features are compressed for classification using MLP classifier.

### eAGCL

**Figure 4:**
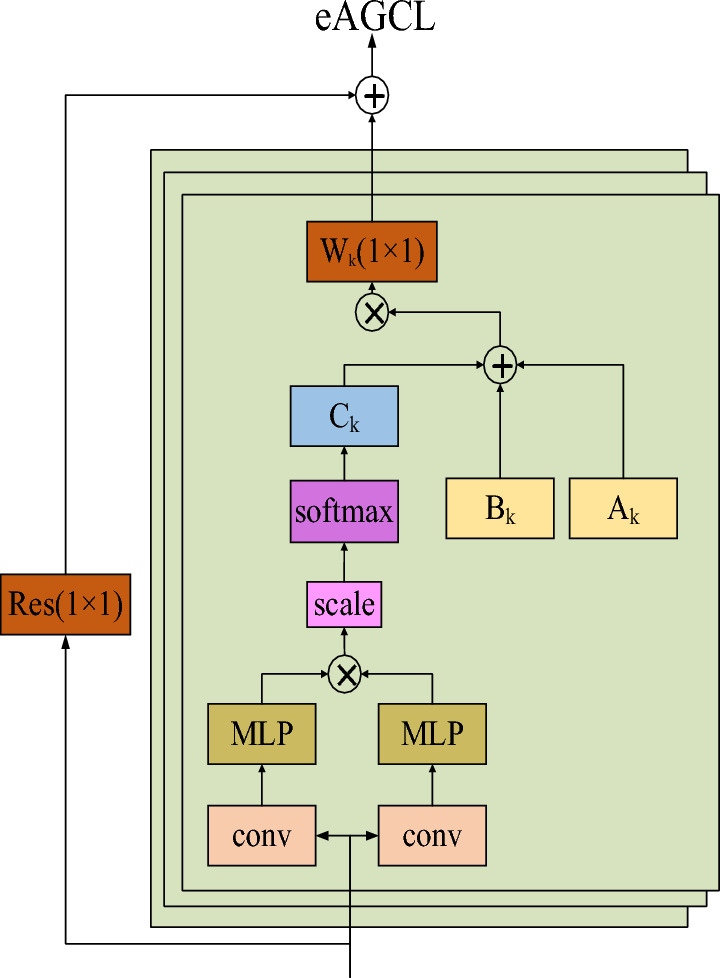
The network structure of eAGCL.

The Internal network structure of eAGCL is described in Fig. 4. We introduce a novel trainable matrix as a replacement for the two Gaussian functions in *C*_*k*_. The trainable matrix facilitates the optimization of network parameters *C*_*k*_ for each sample through back propagation, thereby enhancing the efficiency and effectiveness of learning from sample data. The similarity between joints is computed using a covariance matrix, which is presented in Eq. (3), $$X=\left[\begin{array}{cc}\begin{array}{cc}\overrightarrow{{x}_{0}}& \overrightarrow{{x}_{1}}\end{array}& \begin{array}{cc}\cdots & \overrightarrow{{x}_{n}}\end{array}\end{array}\right]$$ is a d-dimensional eigenvector and ${X}^{T}$ is the transpose of *X*. The covariance matrix can capture the similarity between different dimensions in multiple elements, and the stronger the similarity, the higher the covariance value.

The shape of the skeleton data is $x\in {R}^{C\times V\times T}$, *C* represents the x, y, z coordinates, *V* denotes the number of joints and *T* is the length of the skeleton sequence. To convert the skeleton data into d-dimensional feature vectors, a trainable matrix *conv* is introduced to transform the skeleton data into $\text{x}\in {\text{R}}^{\text{d}\times \text{T}}$, elements in the trainable matrix can be regarded as initial weight values. They are optimized through back propagation. The dot product of each generated feature vector is divided by $\sqrt{\text{d}}$, and we can get the final weighted values using softmax. Expression for *C*_*k*_ is shown in Eq. (4). The value of variance of each element in *C*_*k*_ depends on *d*, thus pushing softmax function into the area with minimal gradient. To counteract this effect, we scale the dot products by $\frac{1}{\sqrt{d}}$.

3 $$\begin{array}{c}{X}^{T}X=\left[\begin{array}{ccc}\begin{array}{cc}{x}_{0}\cdot {x}_{0}& {x}_{0}\cdot {x}_{1}\\ {x}_{1}\cdot {x}_{0}& {x}_{1}\cdot {x}_{1}\end{array}& \begin{array}{c}\cdots \\ \cdots \end{array}& \begin{array}{c}{x}_{0}\cdot {x}_{n}\\ {x}_{1}\cdot {x}_{n}\end{array}\\ \begin{array}{cc}\vdots & \vdots \end{array}& \ddots & \vdots \\ \begin{array}{cc}{x}_{n}\cdot {x}_{0}& {x}_{n}\cdot {x}_{1}\end{array}& \cdots & {x}_{n}\cdot {x}_{n}\end{array}\right]\end{array}$$

4 $$\begin{array}{c}{C}_{k}=softmax\left(\frac{{\text{conv}(\text{x})}^{T}\text{conv}(\text{x})}{\sqrt{\text{d}}}\right)\end{array}$$

5 $$\begin{array}{c}\left\{\begin{array}{c}PE\left(pos,2l\right)=\text{sin}\left(\frac{pos}{{T}^\frac{2l}{n}}\right)\\ PE\left(pos,2l+1\right)=\text{cos}\left(\frac{pos}{{T}^\frac{2l}{n}}\right)\end{array}\right.\end{array}$$

### Positional encoder

Skeleton data is a form of sequential data that necessitates the utilization of a position encoder to augment the temporal positioning information inherent in the skeleton data. However, ViT employs learnable embedding vectors on position encoders without any involvement in supplementing timing information. While the position encoder in NLP will generate significant superfluous positional encoding information when processing skeleton data.

To address this issue, this paper proposes a redefined position encoder as depicted by Eq. (5), PE(*pos*,2 l) denotes the output result of the positional encoder, and *pos* represents the position of the feature vector in skeleton sequence, 2 l and 2 l + 1 indicate its odevity of *pos*, and *n* signifies the dimensionality of skeleton. The sine function is employed when *pos* is odd while cosine function is utilized when *pos* is even, *T* corresponds to the maximum length of the skeleton sequence. In order to capture and retrieve the entire sequence information, the model needs to add a new vector * at the end of the feature vector, which is then used to embed the position encoder value for each frame.

The relative position of the skeleton sequence can be calculated using Eq. (6) when its offset is m (a positive integer). Upon deduction, an elegant dot product formula emerges, which coincides with the standard inner product formula in Euclidean space. This formulation reveals a crucial topological structure that can be described mathematically: the encoded result at any given position can be decomposed into the dot product of two summations.

$$PE\left(pos+m,2l\right)=\text{sin}\left(\frac{pos}{{1000}^\frac{2l}{n}}+\frac{m}{{1000}^{\frac{2l}{\text{n}}}}\right)$$

6 $$ \begin{aligned} = & \cos \left( {\frac{pos}{{1000^{\frac{2l}{n}} }}} \right)sin\left( {\frac{m}{{1000^{{\frac{2l}{{\text{n}}}}} }}} \right) + sin\left( {\frac{pos}{{1000^{\frac{2l}{n}} }}} \right)cos\left( {\frac{m}{{1000^{{\frac{2l}{{\text{n}}}}} }}} \right) \\ = & PE\left( {pos,2l} \right)sin\left( {\frac{m}{{1000^{\frac{2l}{n}} }}} \right) + PE\left( {pos,2l + 1} \right)cos\left( {\frac{m}{{1000^{\frac{2l}{n}} }}} \right){ } \\ { } = & PE\left( {pos,2l} \right)PE\left( {m,2l + 1} \right) + PE\left( {pos,2l + 1} \right)PE\left( {m,2l} \right) \\ = & \left( {{\text{PE}}\left( {\text{pos,2l}} \right){\text{,PE(pos,2l + 1)}}} \right) \odot {\text{(PE}}\left( {\text{m,2l + 1}} \right){\text{,PE}}\left( {\text{m,2l}} \right){)} \\ \end{aligned} $$

**Figure 5:**
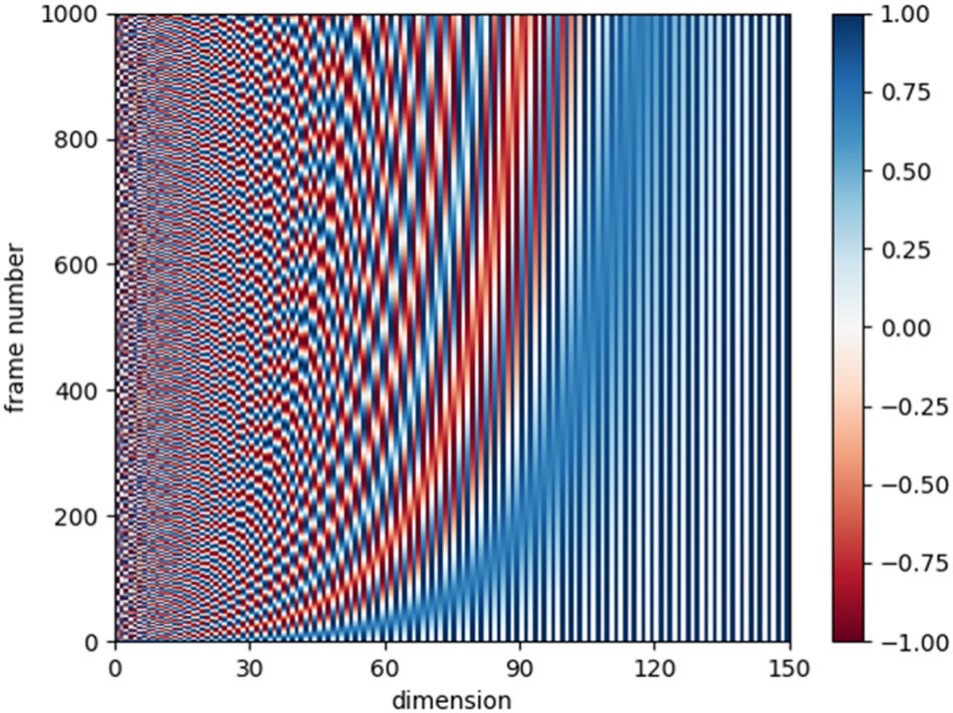
Visualization thermal map of position encoder.

The topology employed facilitates the acquisition of relative position results effortlessly. We quickly obtain its frame sequence by introducing position embedding. Its visual heat map is shown in Fig. 5, where the horizontal coordinate represents the dimension and the vertical coordinate represents the frame number.

Position Encoder structure diagram is illustrated in Fig. 3, where rounded rectangle in blue with frame sequence number represent skeleton data and rounded rectangle in pink describe the positional embedding which corresponding with the skeleton data. We introduce a novel frame embedding vector with “T+1” and a novel positional embedding vector *, aiming to effectively capture and retrieve comprehensive timing sequence information.

### Transformer encoder

**Figure 6:**
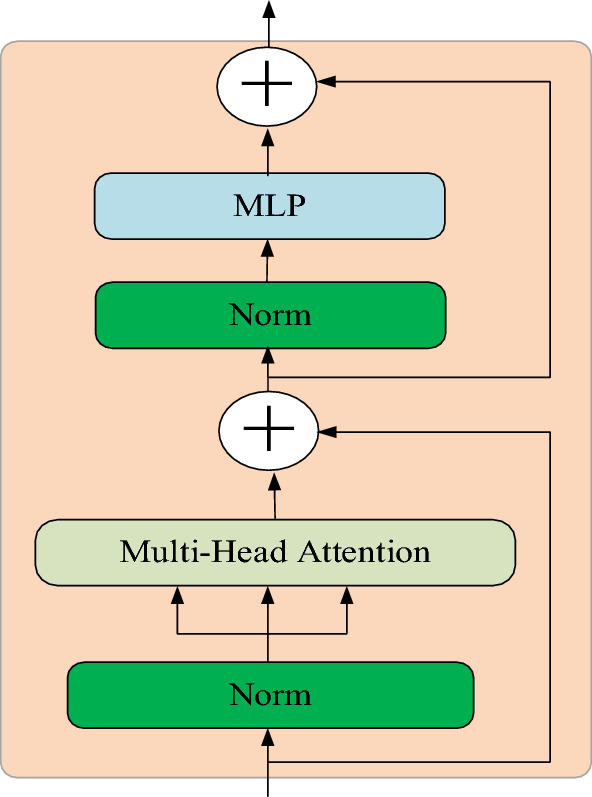
The structure of Transformer Layer.

The Transformer Encoder, comprising MLP and self-Attention modules, is employed to transform input sequences into hidden representations. The initial input consists of the human pose embedding vector for each position, which is then encoded into a fixed-length hidden vector representation through multiple layers of self-attention mechanism and fully connected layers. Figure 6 illustrates the structure of the Transformer Encoder.

### Multi-layer perceptron (MLP) classifier

The multi-layer neural network is represented by Eq. (7), where *W*_*1*_ and *W*_*2*_ denote the weights of the first and second layers respectively, while *b*_*1*_ and *b*_*2*_ represent the biases of the first and second layers correspondingly. Moreover, max(0, x) denotes the ReLU() activation function. It should be noted that through feed forward propagation, this neural network can approximate any continuous or square integrable function with arbitrary precision, thereby enabling accurate classification of any finite training sample set. The model exhibits a classification effect due to the inclusion of feed forward neural networks. Specifically, the frame embedding vector * is extracted from the end of the Transformer compressed coding vector and fed into the MLP classifier, as depicted in Fig. 6.

7 $$\begin{array}{c}FFN\left(x\right)=max\left(0,{W}_{1} X+{b}_{1} \right){W}_{2}+{b}_{2} \end{array}$$

### Ethical approval and informed consent

Data used in our study are publicly available, and ethical approval and informed consent were obtained in each original study.

## Experiments and results

### Skeleton-based action datasets

To demonstrate the effect of the proposed HAR-ViT, four datasets were utilized in this paper: NTU RGB+D 60, NTU RGB+D 120, Kinetics-Skeleton 400 and our homemade data. The brief introduction is as follows.

**NTU RGB**+**D**. It is a large-scale human action recognition dataset. NTU RGB+D 60 contains 56,880 sequences over 60 classes. It provides the 3D Cartesian coordinates of 25 joints, which are captured from 3 Microsoft Kinect v2 cameras with different viewpoints, for each human in an action sample. Each action sample is performed by 40 volunteers in different age groups. NTU RGB+D 120 is an extended version of NTU RGB+D 60 with an additional 60 action classes, with a total of 113,945 sequences. The datasets can be accessed publicly on https://rose1.ntu.edu.sg/dataset/actionRecognition/. We use four benchmarks recommended by the official for a fair comparison with SOTA methods:

1. NTU60 cross-subject (NTU60-Xsub): where the 40 subjects are divided into training and testing groups.
2. NTU60 cross-view (NTU60-Xview):the data from camera views 2 and 3 are used for training, and data from camera view 1 is used for testing.
3. NTU120 cross-subject (NTU120-Xsub):where the 106 subjects are divided into training and testing groups.
4. NTU120 cross-setup (NTU120-Xset): the data from samples with even setup IDs are used for training, and data from samples with odd setup IDs are used for testing.

**Kinetics-Skeleton 400**. The skeleton data includes 18 major joints of the human body. It contains more than 300,000 clips covering 400 action categories. Among 260,000 total samples, 240,000 samples are used for training and 20,000 for testing. The datasets can be accessed publicly on https://deepmind.com/research/open-source/kinetics.

**Homemade datasets**. We utilize 3 cameras with different views to capture 10 action classes, each action is performed from 10 volunteers (in our research laboratory) , it contains 100 videos.

### Experiment description

The baseline and platform details are depicted as:

**Comparison Baseline**: In the experiment, the comparison baseline is the representative 2s-AGCN^13^ consisting of 10 layers of TCN-GCN. To prove the effectiveness of our methods, the training and test samples of this work are consistent with the baseline. No additional training strategies are applied in this work.

**Platform Details**:The experimental setup for this study involves a NVIDIA GeForce RTX 2070 SUPER server running on Ubuntu 20.04.5 LST (CPU: 4 cores, memory: 16 GB, video memory: 8 GB), paddle2.5.1, and cuda11.4. Table 1 presents the training configuration parameters used in HAR-ViT.

**Table 1:** The training configuration parameters of HAR-ViT.

Parameters	Values
Learning rate	10^−3^
Optimizer	Adam
Loss function	Cross entropy

### Training comparison with SOTA methods

We compare the training results with three open source code SOTA methods, including ST-GCN, 2s-AGCN and DSTA-Net.

The number of model parameters of each comparison algorithm is presented in Table 2. It can be observed that the model proposed in this paper achieves a reduction of 57.24% in parameter count compared with 2s-AGCN, while also demonstrating improved efficiency and lightweight characteristics.

**Table 2:** Comparison of parameter number of four methods.

Methods	Parameter ↓ (MB)
ST-GCN^12^	90310.27
2s-AGCN^13^	10694.15
DSTA-Net^31^	8636.53
HAR-ViT	4572.60

#### Experiments on NTU RGB+D 60

**Figure 7:**
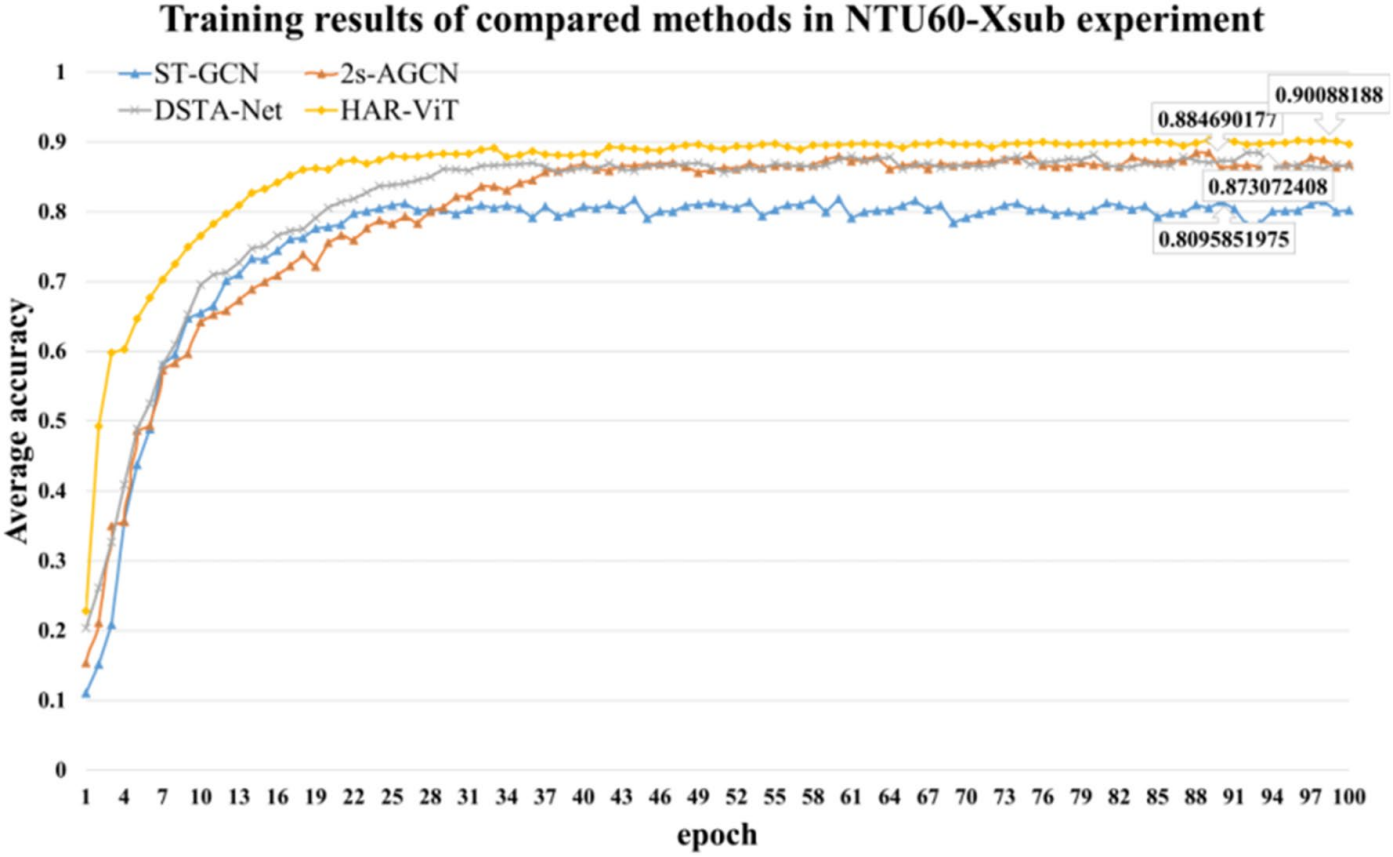
Training results of compared methods in NTU60-Xsub experiment.

In NTU60-Xsub experiment, the training set and testing set are divided in 1:1, ensuring complete independence between the subjects. This is performed to assess the model's capability of recognizing unfamiliar subjects. The training results of each compared algorithm are illustrated in Fig. 7. After the same training epochs, the average accuracy of HAR-ViT is higher than other three methods, and it soon reaches a higher level after 30 training epochs, so the proposed algorithm exhibits a higher fitting speed.

**Figure 8:**
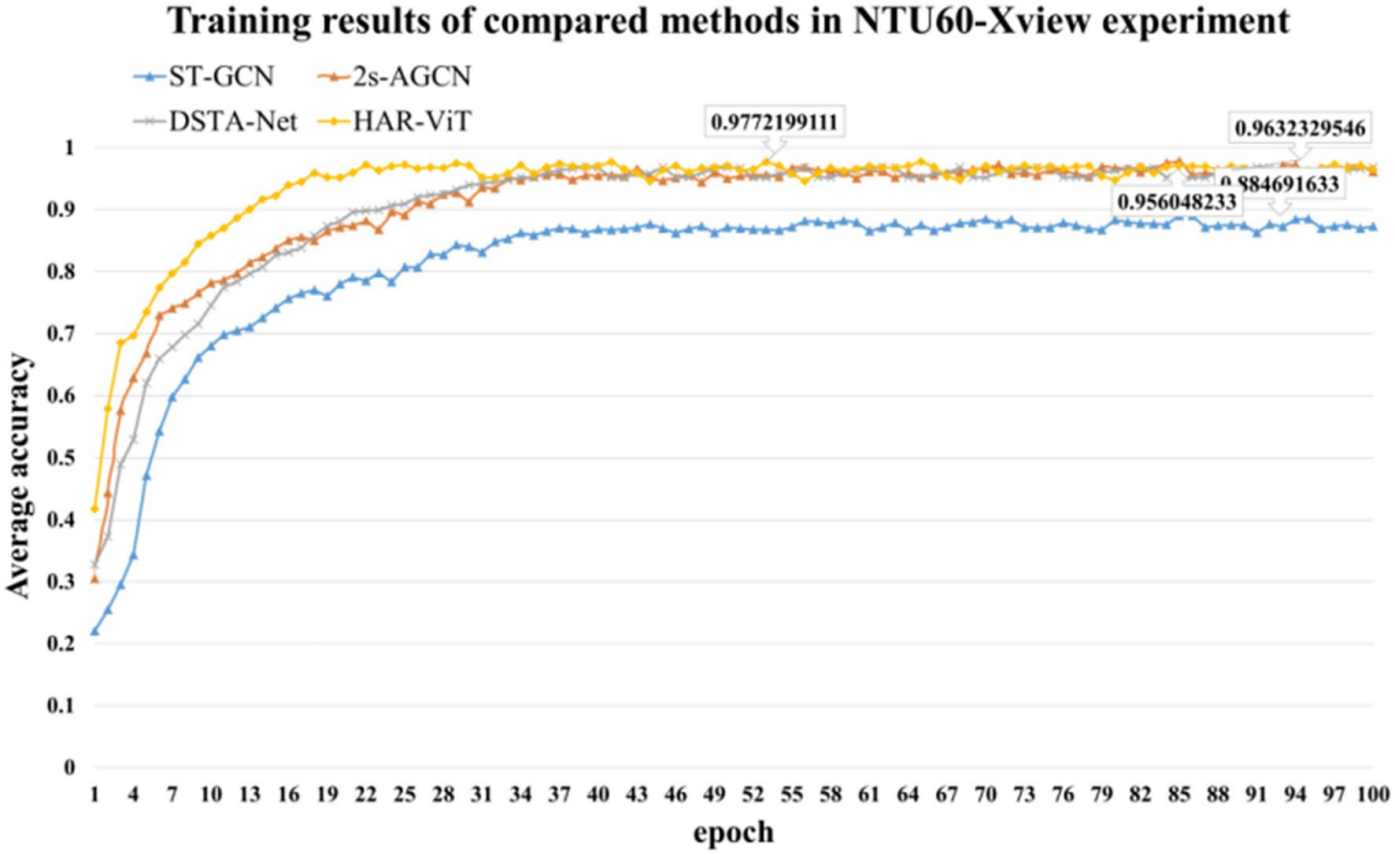
Training results of compared methods in NTU60-Xview experiment.

In NTU60-Xview experiment, the ratio between the training set and the test set is 2:1. Through such experiments, we can conduct a more specific analysis of the model's effectiveness in recognizing unconventional angular actions (view 1) when trained on other views (view 2 and 3). The training results of each comparative algorithm are depicted in Fig. 8. Our proposed model demonstrates exceptional adaptability to diverse shooting angles, exhibiting a notable 5.9% improvement compared to NTU60-Xsub. Moreover, when compared with other algorithms, HAR-ViT showcases a more pronounced enhancement.

#### Experiments on NTU RGB+D 120

**Figure 9:**
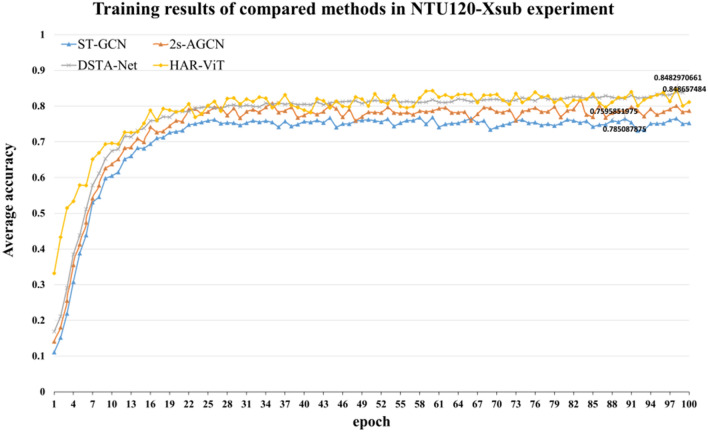
Training results of compared methods in NTU120-Xsub experiment.

In NTU120-Xsub experiment, the ratio between the training set and the test set is 1:1. The results of the four methods are illustrated in Fig. 9. In comparison to NTU RGB+D 60, there is a decrease in average accuracy, which can be attributed to the expanded range of identified categories. Our model demonstrates greater effectiveness when compared with other algorithms.

**Figure 10:**
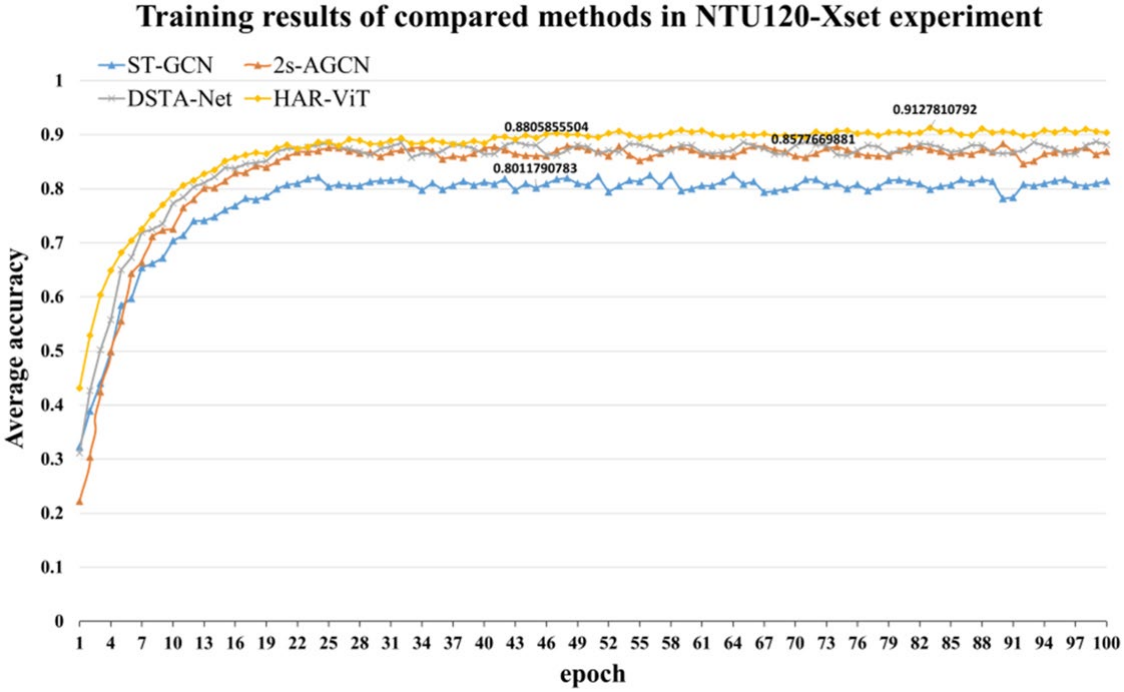
Training results of compared methods in NTU120-Xset experiment.

In NTU120-Xset experiment, the training results of each model are depicted in Fig. 10. We can observe that the accuracy of all the models slightly decreased compared to NTU120-Xsub experiment, but the average accuracy of our model is higher than that of 2s-AGCN and other models. This demonstrates that our method exhibits more pronounced advantages when compared with other algorithms.

#### Experiments on Kinetics-Skeleton 400

**Figure 11:**
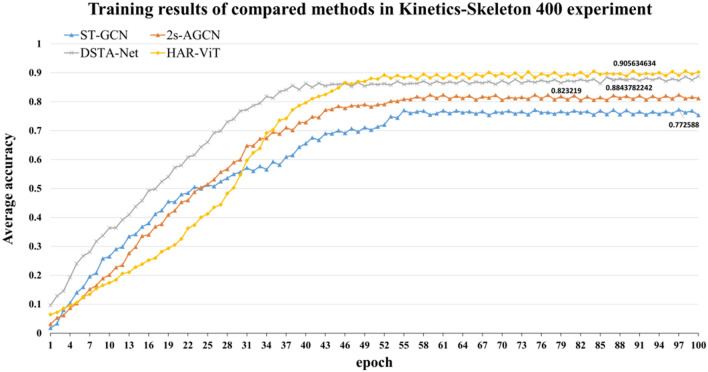
Training results of compared methods in Kinetics-Skeleton 400 experiment.

The training results of each model on Kinetics-Skeleton 400 are described in Fig. 11. We can see that the accuracy of all the models slightly decreased compared to NTU RGB+D, because the number of action categories covered is 3.3 times that of NTU RGB+D. Otherwise, the average accuracy of our model is higher than that of 2s-AGCN and other models. It shows that the algorithm in this paper has stronger generalization ability.

### Test accuracy comparison with SOTA

To demonstrate the effectiveness and advancements of our HAR-ViT, the test recognition performance is compared with SOTA methods, and the visual classification results of the four compared methods using aforementioned trained model in Section "Training comparison with SOTA methods" are also demonstrated.

#### Experiments on NTU RGB+D 60

The comparison on NTU-RGB+D 60 is shown in Table 3, the best results are in bold and the suboptimum results are italics. The recognition accuracy of our HAR-ViT is increased by 2.56% under X-Sub and 1.63% under X-View than 2s-AGCN. The DSTA-Net represents the optimal outcome achieved by X-Sub, effectively extracts temporal and spatial features through the spatio-temporal attention. HAR-ViT exhibits a slight decrease of 0.44%under X-Sub compared with DSTA-Net, potentially due to the equilibrium of spatio-temporal convolution in DSTA-Net. Notably, HAR-ViT outperforms AAM-GCN and LCK-GCN by 0.66% and 0.36% respectively, simulating remote features using attention mechanisms as in 2s-AGCN.

**Table 3:** Comparison of accuracy of nine methods on NTU-RGB+D 60.

Methods, proposed year	NTU RGB+D 60↑(%)	
	X-sub	X-view
ST-GCN, 2018^12^	81.5	88.3
2s-AGCN, 2018^13^	88.5	95.1
AGCN, 2019^14^	88.0	95.1
AS-GCN, 2019^29^	86.8	94.2
CA-GCN, 2020^15^	86.5	94.1
Shift-GCN, 2020^30^	90.7	*96.5*
DSTA-Net, 2020^31^	**91.5**	96.4
AAM-GCN, 2021^32^	90.4	96.2
SS-GCN, 2021^16^	83.6	90.3
LKA-GCN, 2023^19^	90.7	96.1
HAR-ViT, 2023	*91.06*	**96.73**

The performance of Shift-GCN is sub-optimal under X-view, with HAR-ViT outperforming it by 0.23% under X-view. The incorporation of the Shift graph convolution operation and lightweight point convolution in Shift-GCN enhances its spatial feature extraction capability. However, HAR-ViT's eAGCL effectively adapts to skeleton data through trainable matrices, mitigating the impact of positional errors and yielding superior results compared to Shift-GCN. Under X-view, HAR-ViT achieves improvements of 0.33% and 0.43%over AAM-GCN and DSTA-Net respectively. AAM-GCN exhibits limited generalization ability when confronted with different angles, while DSTA-Net lacks a fixed skeleton graph in its spatial attention mechanism. In contrast, eAGCL within HAR-ViT maintains a consistent skeleton diagram and ensures model stability through residual connections, leading to better performance across different shooting angles compared to other algorithms.

#### Experiments on NTU RGB+D 120

The comparison on NTU-RGB+D 120 of 6 methods is illustrated in Table 4, the best results are in bold and the sub-optimum results are italics. The recognition performance of our method is 5.81% under X-Sub and 4.12% under X-Set higher than the baseline 2s-AGCN.

**Table 4:** Comparison of accuracy of nine methods in cross-view experiment.

Methods, proposed year	NTU RGB+D 120↑(%)	
	X-sub	X-set
ST-GCN, 2018^12^	76.5	81.5
2s-AGCN, 2018^13^	81.8	84.9
Shift-GCN, 2020^30^	85.9	87.6
DSTA-Net, 2020^31^	*86.6*	*89.0*
LKA-GCN, 2023^19^	86.3	87.8
HAR-ViT, 2023	**87.61**	**89.02**

The accuracy of our method is 1.01% and 0.02% higher than that of the sub-optimal DSTA-Net under X-Sub and X-Set. DSTA-Net expands the receptive field through the self-attention mechanism and achieves excellent recognition performance. However, the feature decoupling strategy of DSTA-Net involves four streams, which leads to expensive computation in case of large-scale sample. In contrast, our HAR-ViT achieves similar performance using only one stream .

#### Top 5 test results

**Figure 12:**
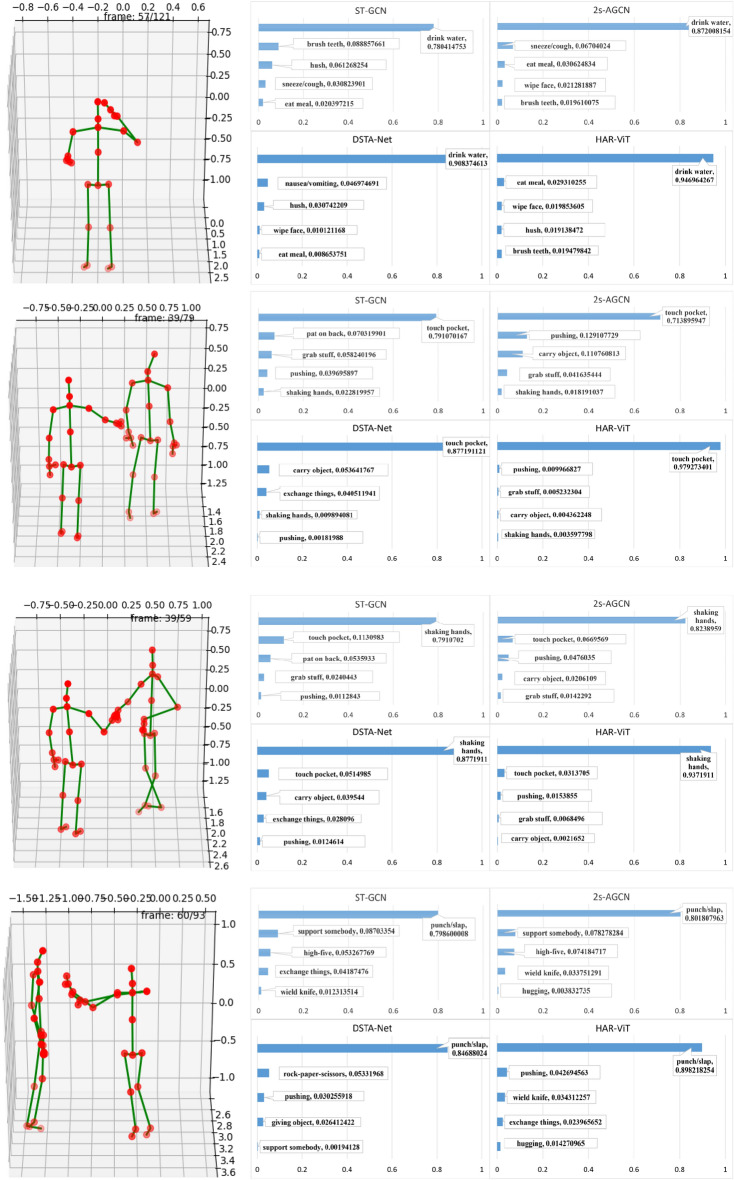
The classification results of actions “drinking water”, “touch pocket”,“shaking hands” and “punch/clap”.

The classification performance of the four methods of four actions “drinking water”, “touch pocket”,“shaking hands” and “punch/clap” on NTU RGB+D are shown in Fig. 12. All four methods correctly identify the four actions, notably, our method exhibits higher confidence than the baseline 2s-AGCN for all the four actions and demonstrates an impressive average confidence level of 94%, while the confidence of 2s-AGCN is 80%. The confidence of ours is the highest among the all the four methods, the validity of our method is illustrated again.

The average inference time of the four algorithms in the test data of the standard dataset is presented in Table 5, with optimal results highlighted in bold and sub-optimal results italics. HAR-ViT achieves an average inference time of 4.75 s, which is 3.5 s faster than both 2s-AGCN and DSTA-Net. This improvement can be attributed to HAR-ViT's reduction in parameter count and substitution of exponential Gaussian function calculations with matrix multiplication, thereby reducing complexity in inference computations.

**Table 5:** Mean inference time on the standard datasets.

Methods	Times↓
ST-GCN^12^	8.75 s
2s-AGCN^13^	9.50 s
DSTA-Net^31^	*8.25 s*
HAR-ViT	**4.75 s**

#### Experiments on Kinetics-Skeleton 400

The comparison on Kinetics-Skeleton 400 is shown in Table 6, the best results are in bold and the sub-optimum results are italics. Our HAR-ViT also achieves excellent performance promotions (+ 2.0% under Top-1 and + 2.2% under Top-5) over the baseline 2s-AGCN. The recognition accuracy is 0.3% higher than LKA-GCN and AGCN under Top-1. Under Top-5, ours is also 0.4% higher than AAM-GCN. It can be found that the performance of AGCN is better than our method and it utilizes multi-stream branch structure and has strong generalization ability.

**Table 6:** Comparison on Kinetics-Skeleton 400 dataset.

Methods, proposed year	Top-1 ↑ (%)	Top-5 ↑ (%)
ST-GCN, 2018^12^	30.7	52.8
2s-AGCN, 2018^13^	36.1	58.7
AGCN, 2019^14^	*37.8*	**61.0**
AS-GCN, 2019^29^	34.8	56.5
CA-GCN, 2020^15^	34.1	56.6
AAM-GCN, 2021^32^	37.5	60.5
SS-GCN, 2021^16^	35.2	57.6
LKA-GCN, 2023^19^	*37.8*	*60.9*
HAR-ViT, 2023	**38.1**	*60.9*

**Figure 13:**
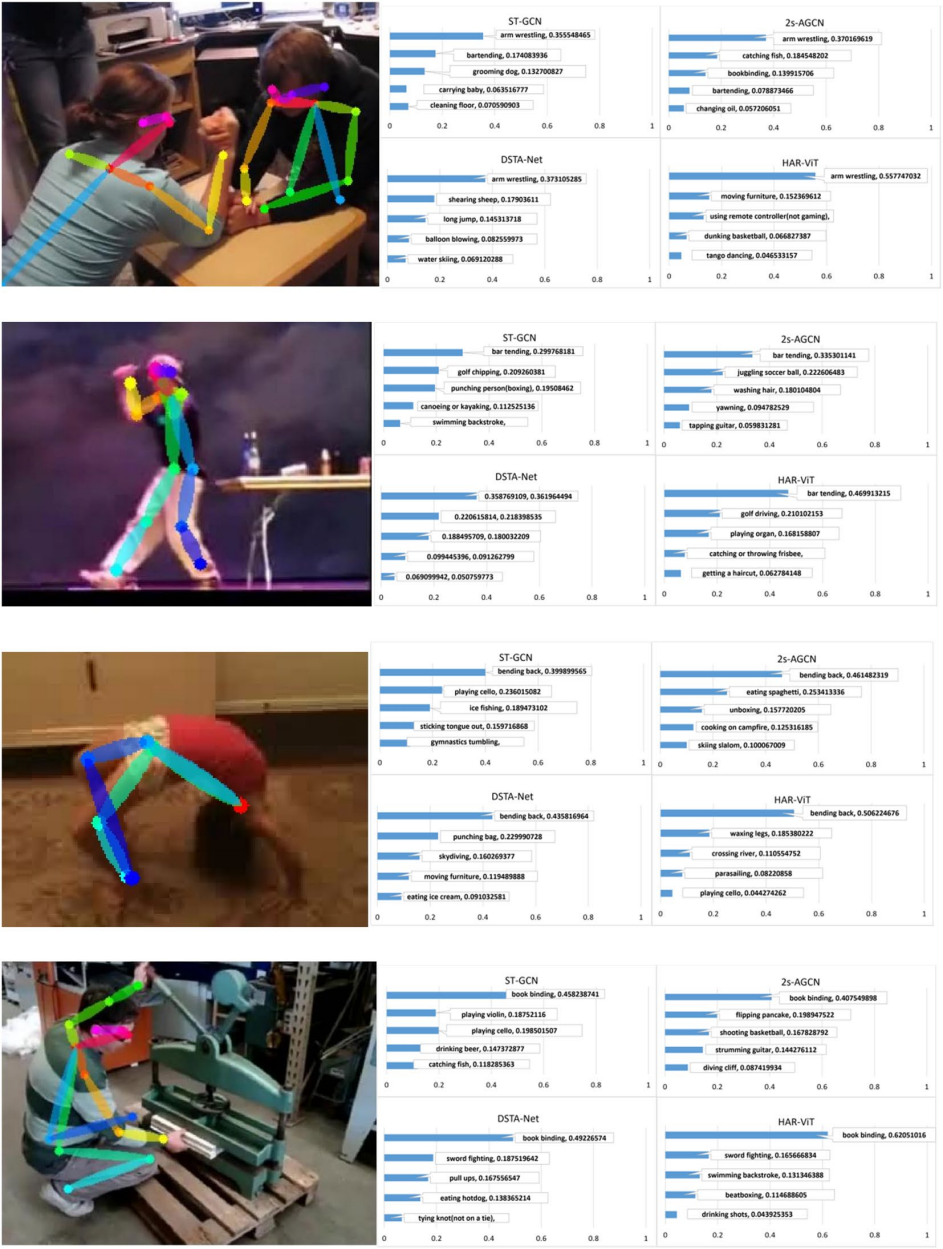
The classification results of actions “arm wrestling”, “bar tending”,“bending back” and “book binding”.

The classification performance of the four methods of four actions “arm wrestling”, “bar tending”,“bending back” and “book binding” under Top-5 on Kinetics-Skeleton 400 are shown in Fig. 13. All four methods correctly identify the four actions, notably, our method exhibits higher confidence than the baseline 2s-AGCN for all the four actions and demonstrates an impressive average confidence level of 54%, while the confidence of 2s-AGCN is 39%. Because the dataset covers far more classes than NTU RGB+D, the confidence level drop significantly for all the four methods.

### Experiment on real-world datasets

**Figure 14:**
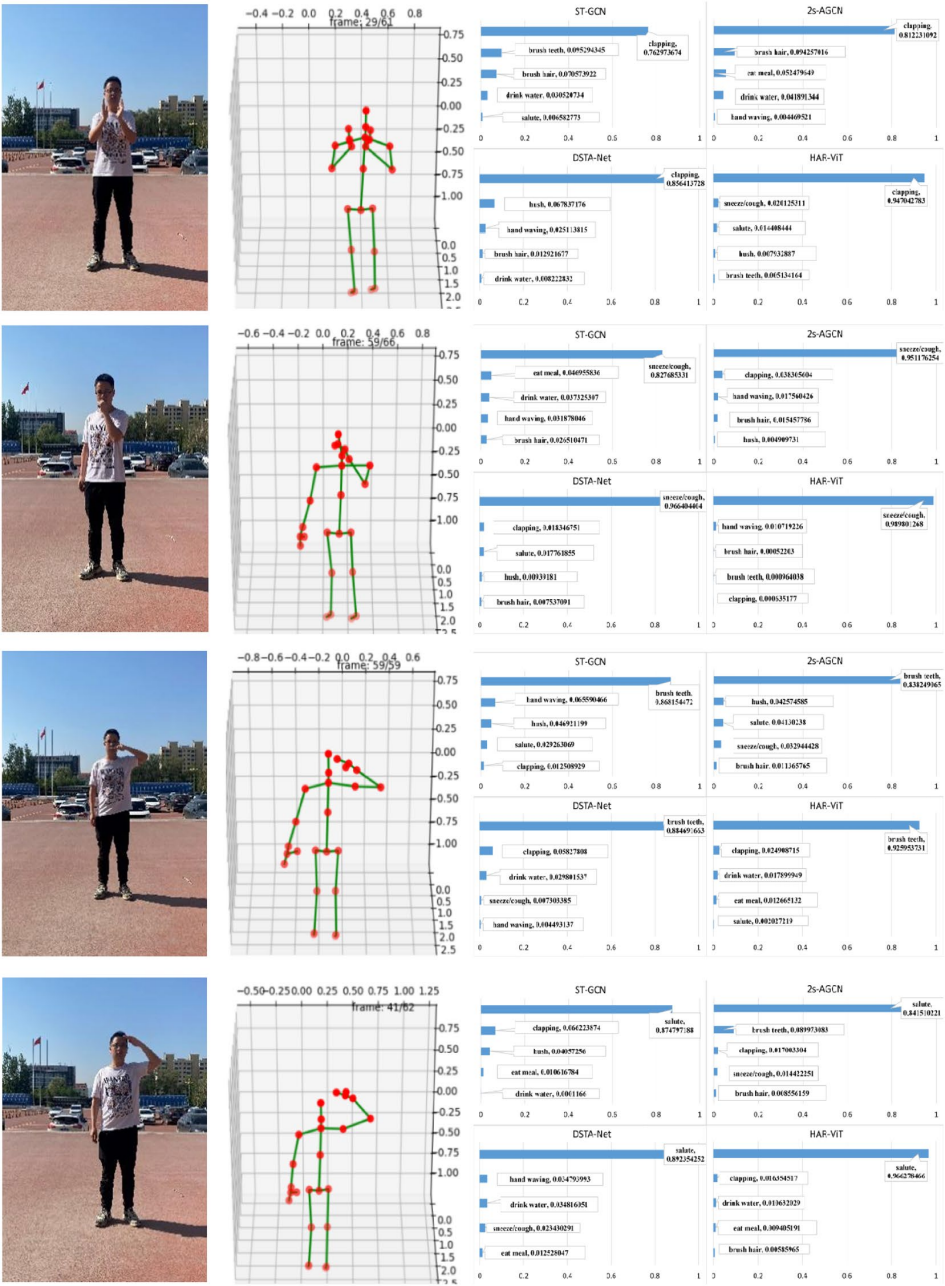
The classification result of action “clapping”, “brush teeth”,“sneeze/cough” and “salute”.

In order to prove the generalization ability of our algorithm, we also test it on homemade datasets in addition to widely used standard datasets. The classification results of four action “clapping”, “brush teeth”,“sneeze/cough” and “salute” are shown in Fig. 14. We can see that four methods can recognize all the four actions positively, however, our method exhibits higher confidence than all the other three methods even for the three similar actions( “brush teeth”,“sneeze/cough” and “salute” ). ours demonstrates an impressive average confidence level of 96%, while the confidence of 2s-AGCN is 86%.

Table 7 presents the average inference time of the four algorithms on the homemade datasets. The optimal results are highlighted in bold, while the sub-optimal results are italics. HAR-ViT demonstrates an average inference time that is 4 s and 3 s faster than 2s-AGCN and DSTA-Net, respectively. Since the skeleton data in the real environment consists of single action, its inference time is significantly shorter compared to that of standard datasets in Table 6.

**Table 7:** Mean inference time on the homemade datasets.

Methods	Times↓
ST-GCN^12^	7.75 s
2 s-AGCN^13^	8.25 s
DSTA-Net^31^	*7.25 s*
HAR-ViT	**4.25 s**

### Ablation experiment

In order to validate the efficacy of each module in our proposed method, we conducts ablation experiments. HAR-ViT model combines the strengths of 2s-AGCN and ViT models. However, it should be noted that 2s-AGCN has the difficulty of gradient disappearance and unsubstantial capturing in hidden data. Hence, we have made enhancements to 2s-AGCN, namely eAGCL, and it serves as the baseline, the results of the ablation experiment are presented in Table 8. We can include that the each model in our method plays an important role. By incorporating eAGCL, ViT's applicability is extended from 2D images to 3D skeleton information. Additionally, the introduction of a position encoder enables ViT to specialize in time series data. Transformer encoder efficiently compresses sequence data features to enhance calculation speed.

**Table 8:** Ablation experiment results of our method.

Models	Transformer	Position	Accuracy ↑(%)
eAGCL	×	×	70.6
eAGCL+position encoder	×	√	79.6
eAGCL+transformer encoder	√	×	88.3
HAR-ViT	√	√	94

Increasing the depth of the Transformer encoder leads to excessive complexity and overfitting of the model. Ablation experiments of the depth of Transformer encoder are shown in Table 9, it is demonstrated that depth of 12 achieves optimal performance, and this configuration was adopted in all experiments in this paper.

**Table 9:** Ablation experiment results of the depth of transformer encoder.

The depth of Transformer encoder	Accuracy ↑(%)
3	67.18
6	82.58
9	84.97
12	90.08
15	86.21

## Conclusions

The paper presents HAR-ViT, a novel method for human activity recognition. X-sub, X-view and X-set experiments on standard widely datasets demonstrate that the HAR-ViT model exhibits fast training speed and requires fewer calculation parameters. Mathematical analysis and test experimental results further confirm that HAR-ViT can achieve SOTA performance and have higher accuracy than some GCN-basd methods.

However, we also found some limitations. On three skeleton-based action datasets, this work demonstrates the model prototype and provides a clear plan for further study, but doesn’t test on other specific action datasets. In the future, the scope of our research will widen to include other forms of data, including depth maps and point cloud. Furthermore, our method cannot solve the incomplete skeleton data, because the lack of any joint may lead to inaccurate attention results. Therefore, it is also to be studied in the future.

## Data Availability

The datasets generated during and/or analyzed during our study are available from the corresponding author on reasonable request.
